# Age-Specific ^18^F-FDG Image Processing Pipelines and Analysis Are Essential for Individual Mapping of Seizure Foci in Pediatric Patients with Intractable Epilepsy

**DOI:** 10.2967/jnumed.117.203950

**Published:** 2018-10

**Authors:** Bianca De Blasi, Anna Barnes, Ilaria Boscolo Galazzo, Chia-ho Hua, Barry Shulkin, Matthias Koepp, Martin Tisdall

**Affiliations:** 1 Department of Medical Physics, University College London, London, United Kingdom; 2 Institute of Nuclear Medicine, University College London Hospitals, London, United Kingdom; 3 Department of Computer Science, University of Verona, Verona, Italy; 4 Department of Radiation Oncology, St. Jude Children’s Research Hospital, Memphis, Tennessee; 5 Department of Diagnostic Imaging, St. Jude Children’s Research Hospital, Memphis, Tennessee; 6 Institute of Neurology, University College London, London, United Kingdom; and; 7 Great Ormond Street Hospital, London, United Kingdom

**Keywords:** pediatric ^18^F-FDG PET template, voxelwise statistics, epilepsy

## Abstract

^18^F-FDG PET is an important tool for the presurgical assessment of children with drug-resistant epilepsy. Standard assessment is performed visually and is often subjective and highly user-dependent. Voxelwise statistics can be used to remove user-dependent biases by automatically identifying areas of significant hypo- or hypermetabolism associated with the epileptogenic area. In the clinical setting, this analysis is performed using commercially available software. These software packages suffer from two main limitations when applied to pediatric PET data: pediatric scans are spatially normalized to an adult standard template, and statistical comparisons use an adult control dataset. The aim of this work was to provide a reliable observer-independent pipeline for the analysis of pediatric ^18^F-FDG PET scans, as part of presurgical planning in epilepsy. **Methods:** A pseudocontrol dataset (19 subjects 6–9 y old, and 93 subjects 10–20 y old) was used to create two age-specific ^18^F-FDG PET pediatric templates in standard pediatric space. The ^18^F-FDG PET scans of 46 epilepsy patients (16 patients 6–9 y old, and 30 patients 10–17 y old) were retrospectively collated and analyzed using voxelwise statistics. This procedure was implemented with the standard pipeline available in the commercial software Scenium and an in-house Statistical Parametric Mapping, version 8 (SPM8), pipeline (including age-specific pediatric templates and reference database). A κ-test was used to assess the level of agreement between the findings of voxelwise analyses and the clinical diagnosis of each patient. The SPM8 pipeline was further validated using postsurgical seizure-free patients. **Results:** Improved agreement with the clinical diagnosis was reported using SPM8, in terms of focus localization, especially for the younger patient group: κ = 0.489 for Scenium versus 0.826 for SPM. The proposed pipeline also showed a sensitivity of about 70% in both age ranges for the localization of hypometabolic areas on pediatric ^18^F-FDG PET scans in postsurgical seizure-free patients. **Conclusion:** We showed that by creating age-specific templates and using pediatric control databases, our pipeline provides an accurate and sensitive semiquantitative method for assessing the ^18^F-FDG PET scans of patients under 18 y old.

Multimodal imaging in patients with drug-resistant epilepsy is used to guide presurgical assessment in potential candidates for surgery (1). This is a challenging procedure that aims to precisely localize the epileptogenic focus and its relation to the surrounding cortex and thus direct subsequent surgical resection. Although ^18^F-FDG PET has been used since the 1980s to detect hypometabolism associated with the epileptogenic zone (2,3), several methodologic constraints limit its use, particularly in the pediatric population.

^18^F-FDG PET data are routinely assessed visually by expert readers. This qualitative evaluation is subjective and highly user-dependent, it might miss subtle or bilateral intensity changes, and its effectiveness varies with epilepsy type and focus location (2). More sophisticated voxelwise statistical methods can be used to remove user-dependent biases, thereby improving reproducibility and preventing disagreement arising from visual assessment of PET scans (2). These methods spatially normalize the patient brain scan to a common standard template, where it is compared with a normative dataset. This voxelwise comparison leads to the automatic identification of areas of hypo- or hypermetabolism at the individual level. In adults (above 18 y old), voxelwise statistics have proven robust and reliable (4–6). In pediatric patients, the efficacy of this analysis remains less clear because of age-related variations in brain size and metabolism. Only a few studies have used age-matched pediatric databases for statistical comparison, and a detailed description of a pediatric ^18^F-FDG PET template for spatial normalization is still lacking (2,7).

Voxelwise analysis is usually performed using Food and Drug Administration–approved automated software packages provided, for example, by Siemens (Scenium) or GE Healthcare (CortexID). Despite being user-friendly and highly intuitive, these software applications suffer from two main limitations when applied to pediatric PET data: the spatial normalization of the pediatric scans to adult Montreal Neurological Institute (MNI) standard space (8), which leads to unwanted deformations and marked anatomic mismatches between the different brain structures, and the use of an adult inbuilt normative database for statistical comparisons, which differs from children in both brain size and metabolism.

In this study, we investigated whether more accurate and reliable results can be achieved using age-specific templates and databases of ^18^F-FDG PET images, and we introduced a processing pipeline to improve the accuracy with which the epileptogenic zone is localized on pediatric ^18^F-FDG PET scans. Our aim was to create ^18^F-FDG PET templates representing two age ranges (6–9 y and 10–20 y) and use age-appropriate control datasets for voxelwise analysis. This pipeline was quantitatively compared against the commercial software Scenium, currently adopted in clinical settings, and was further validated in postsurgical seizure-free patients, whose clinical follow-up diagnosis was used as the gold standard.

## MATERIALS AND METHODS

The adopted methodology included the voxelwise analysis of a pediatric patient dataset using a commercial software and an ad-hoc pipeline based on the Statistical Parametric Mapping, version 8 (SPM8, http://www.fil.ion.ucl.ac.uk/spm/). For the latter, we built two age-specific ^18^F-FDG PET templates in the pediatric standard space defined by MRI templates, available from the pediatric atlas (9). In the SPM8 pipeline, the patients’ scans were compared with an age-matched pseudocontrol database (i.e., without central nervous system diseases).

### Study Population

The patient dataset was retrospectively collated from Great Ormond Street Hospital and included ^18^F-FDG PET/CT scans of 46 children (16 patients 6–9 y old and 30 patients 10–17 y old) with focal and generalized epilepsies, which reflects what is normally found in presurgical assessments (patient demographics are shown in Supplemental Table 1, available at http://jnm.snmjournals.org). Of these patients, 18 (4 patients 6–9 y old and 14 patients 10–17 y old) underwent surgical resection of the hypothesized epileptogenic area, with the known surgical outcome reported on average 11 mo after surgery (time range of follow-up reports, 1–38 mo). Preoperative MRI scans were collected for all these patients, and postsurgery scans (at least 6 mo of follow-up) were available for 13 patients (4 patients 6–9 y old and 9 patients 10–17 y old).

**FIGURE 1.**
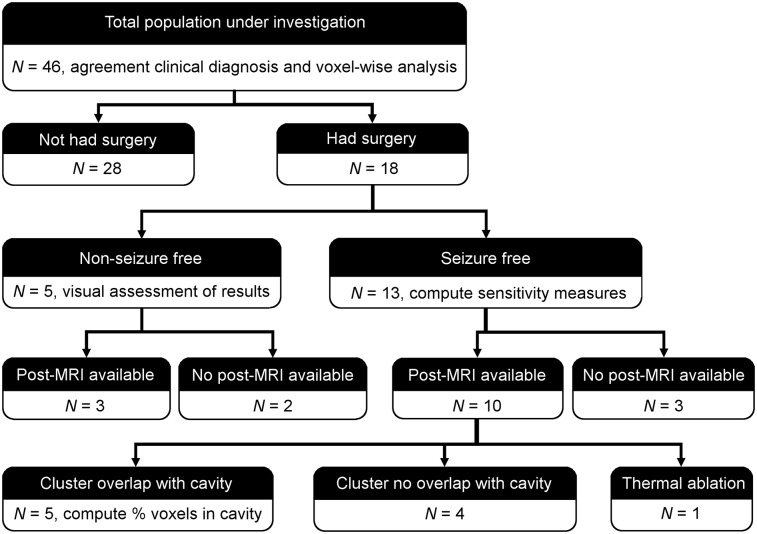
Population under investigation and corresponding analyses for validating SPM8 pipeline.

Figure 1 summarizes the patient dataset under investigation and the corresponding analyses performed. A control dataset (112 subjects 6–20 y old) was also included in the study, as previously described (10). The research and development board at Great Ormond Street Hospital approved this retrospective study, and the requirement to obtain informed consent was waived.

### ^18^F-FDG PET Acquisition

The patient dataset included dedicated ^18^F-FDG PET/CT brain scans of patients who had fasted 4 h before undergoing scanning. ^18^F-FDG was administered on the basis of patient weight (dose range, 14–200 MBq), 30 min before scanning. ^18^F-FDG PET/CT images were acquired for 15 min using a Discovery 710 system (GE Healthcare). Attenuation-corrected PET images were iteratively reconstructed by standard vendor-provided software, using a voxel size of 2 × 2 × 3.27 mm.

The control dataset comprised whole-body ^18^F-FDG PET/CT scans of patients referred for oncology staging but displaying no brain pathology (pseudocontrols) (10). We considered only the brain sections of these scans. ^18^F-FDG PET/CT scanning of these data was performed using a Discovery 690 PET/CT system (GE Healthcare) at multiple bed positions in 3-dimensional mode for 3–5 min per bed position (10). Attenuation-corrected PET images were iteratively reconstructed by standard vendor-provided software, using a voxel size of 3.65 × 3.65 × 3.27 mm. A complete description of the acquisition protocol and parameters was previously published (10).

### Creation of Pediatric Templates

The first objective of this study was to create age-specific pediatric ^18^F-FDG PET templates in standard pediatric space and use them as a reference for spatially normalizing each scan before statistical analysis. Two asymmetric T1-weighted MRI standard templates of 112 children between the ages of 7 and 11 y and 108 children between the ages of 13 and 18.5 y from the pediatric atlas were used (9). These structural templates defined the standard pediatric space on which to build the corresponding age-matched ^18^F-FDG PET templates. The ^18^F-FDG PET pseudocontrol dataset was divided into two groups: 19 children aged 6–9 y and 93 aged 10–20 y. The 10-y-old cutoff was based on the assumption that at this age, brain glucose metabolism starts decreasing toward adult levels (2). The following steps were performed in FSL (https://fsl.fmrib.ox.ac.uk/fsl/fslwiki/) for the two age groups separately. The control scans were registered to the corresponding T1-weighted MRI template (affine registration, 12 degree of freedom [dof]) with mutual information as the cost function (Registration1) (11). The resulting registrations were visually assessed, using several anatomic landmarks as a reference. Successful registrations were those for which the PET image was completely and precisely overlaid on the MR image, especially over the frontal and temporal lobes. Then, an average of the correctly registered PET scans (Average1) was computed. A second registration was performed to register all the control PET scans to Average1 (affine registration, 12 dof), with the correlation ratio as the cost function (Registration2). The goodness of fit of Registration2 was again visually assessed for each control scan. An average of the correctly registered PET scans (Average2) was finally computed. The resulting two PET templates (one per age range) were considered for the spatial normalization of all corresponding age-matched scans before voxelwise statistics.

### Voxelwise Statistical Analysis

**FIGURE 2.**
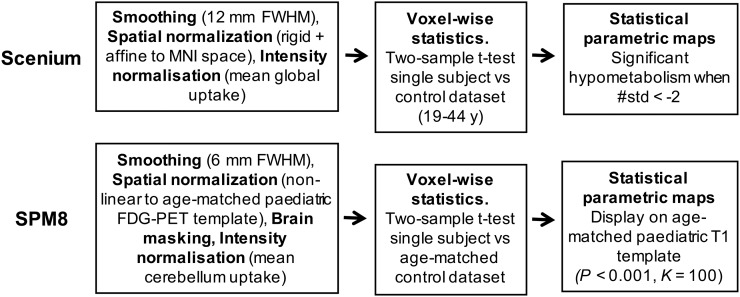
Preprocessing and statistical analysis steps using Scenium and SPM8.

Voxelwise analysis produces statistical parametric maps highlighting regions on a patient’s scan with metabolism significantly different from controls (2). This analysis was performed using Scenium, a Food and Drug Administration–approved, fully automated software program currently available in clinical settings (syngo.via platform, version 11; Siemens) and predominantly used for the presurgical evaluation of epilepsy patients by comparing them with an inbuilt adult control dataset (using ^18^F-FDG PET on a Siemens Biograph scanner, 19–44 y old) (12). Single-subject voxelwise analysis was also performed using a customized pipeline based on SPM8, an open-source suite of MATLAB-based routines in which our in-house pediatric ^18^F-FDG PET templates and control database could be easily integrated. The two pipelines and related processing steps are reported in Figure 2.

Overall, both pipelines included smoothing of the patients’ scans (12-mm fixed kernel in Scenium and 6-mm kernel in SPM8), spatial normalization to standard space (inbuilt adult MNI 91 × 109 × 91, 2-mm isotropic in Scenium; pediatric MNI 197 × 233 × 189, 1-mm isotropic in SPM8), and subsequent intensity normalization (by the mean global uptake in Scenium with limited possibility of change, and by the cerebellum gray matter uptake (3) in SPM8). A two-sample *t* test comparing a single subject with the control group was performed. In Scenium, this testing led to statistical maps, which were displayed as color maps, stereotactic projections, or coronal views. An actual quantification of these results was given by statistics tables showing the number of SDs away from the mean (control uptake) for a standard list of brain regions (Harvard Brain Atlas, keeping the right and left hemispheres separate, 10 regions each). We took as significant those areas displaying SDs of less than −2 (95% confidence interval), that is, reduced glucose metabolism compared with controls. In SPM8, the statistical maps were displayed using a *P* value of less than 0.001 (uncorrected) and a cluster extent of 100.

### Comparison with Clinical Diagnosis

The agreement between the results from the voxelwise analyses (Scenium/SPM8) and the clinical diagnosis was assessed by a neurosurgeon with 10 y of experience in pediatric epilepsy. This assessment was performed by comparing the results of Scenium/SPM8 with the corresponding clinical diagnosis from the multidisciplinary team (MDT), established on the basis of clinical information, electrophysiologic findings, and imaging findings. In the case of focal epilepsies, the area considered was the one with the highest negative *z* score (Scenium) and the one showing the lowest peak-level *P* value (SPM8). We considered as generalized those cases in which more than three regions in different lobes were detected as significant on either of the statistical maps. A κ-test was performed to assess the level of agreement between each pipeline and the MDT diagnosis when focus lateralization (right, left, normal, multifocal) or localization (frontal, temporal, parietal/occipital, normal, multifocal) was considered. Slight agreement was defined as a κ-value of 0.01–0.20, fair agreement as 0.21–0.40, moderate agreement as 0.41–0.60, substantial agreement as 0.61–0.80, and almost perfect agreement as 0.81–0.99 (13).

### Validation Using Postsurgical Patients

To further validate the ad-hoc pipeline based on SPM8, we considered a subgroup of 18 patients (4 patients 6–9 y old and 14 patients 10–17 y old) who underwent surgical resection of the hypothesized focus and had a known outcome. This outcome was defined following the Engel classification, in which a seizure-free outcome corresponds to class I and a non–seizure-free outcome to classes II–IV. Patients who were seizure-free after surgery (3 patients 6–9 y old and 10 patients 10–17 y old) were considered the gold standard (localization of the epileptogenic area could be accurately ascertained) and were included in the following analysis. Postsurgery scans were available for 10 patients (3 patients 6–9 y old and 7 patients 10–17 y old, Fig. 1).

Each patient was analyzed using the aforementioned SPM8 pipeline, and the resulting statistical maps were coregistered to the corresponding presurgical MRI scan, together with the ^18^F-FDG PET and postsurgical MRI scans. Then, the correctness of the lateralization and localization of the statistical maps to the resection cavity was assessed. Lateralization (or localization) was considered to be correct when the cluster containing the maximum on the statistical map fell within the hemisphere ipsilateral to the resected area (in the resected area). Sensitivity was separately defined for lateralization and localization as the proportion of patients in whom the maximum correctly lateralized or colocalized with the resected area. In patients for whom postsurgical MRI scans were not available (3 patients 10–17 y old), the statistical maps were coregistered to the presurgical MRI scan, and lateralization and localization were visually assessed by the same neurosurgeon using surgery reports.

To provide a further quantification of the degree of overlap, the percentage of voxels in the most significant cluster that were falling within the resected area was calculated (Fig. 1). The lesion was segmented out from the postsurgical MRI scan of each patient, using ALI toolbox (http://www.fil.ion.ucl.ac.uk/spm/ext/#ALI) customized with pediatric templates and tissue priors. This lesion segmentation was used to mask the statistical map to calculate the proportion of voxels falling within the resected area.

In the five non–seizure-free patients (1 patient 6–9 y old and 4 patients 10–17 y old), our findings were visually compared with the area resected to evaluate the ^18^F-FDG PET results (Fig. 1).

## RESULTS

### Creation of Pediatric Templates

The first intermodal registrations (source: control scans; target: age-matched T1-weighted MRI pediatric standard space) led to only 8 of 19 (42%, 6–9 y old) and 22 of 93 (24%, 10–20 y old) optimally aligned PET scans that were used to compute Average1. This initial pediatric PET template increased the accuracy of the subsequent registrations, so that the second intramodality registrations (source: control scans ; target: Average1) resulted in an increased number of perfectly aligned scans used to create Average2: 14 of 19 (74%) and 41 of 93 (44%).

### Comparison of Scenium and SPM8

The results of the Scenium/SPM8 pipelines were compared with the MDT diagnosis available for each patient (patient diagnoses, and results of voxelwise analyses are shown in Supplemental Table 2). Table 1 reports the results of the κ-test for both Scenium and SPM8. Overall, Scenium showed substantial agreement with the MDT diagnosis for lateralization and only moderate agreement for localization. Conversely, SPM8 achieved an overall substantial agreement for both lateralization and localization. SPM8 showed an almost perfect agreement for localization in the younger group (0.826), whereas Scenium showed the lowest agreement, in the moderate range (0.489).

**TABLE 1:** κ-Test Results Quantifying Level of Agreement (Lateralizing and Localizing) Between Each Pipeline and MDT Diagnosis for Each Patient

	Lateralization		Localization	
Patients	Scenium	SPM	Scenium	SPM
All (*n* = 46)	0.680	0.770	0.536	0.734
6–9 y old (*n* = 16)	0.630	0.805	0.489	0.826
10–17 y old (*n* = 30)	0.705	0.752	0.555	0.684

**FIGURE 3.**
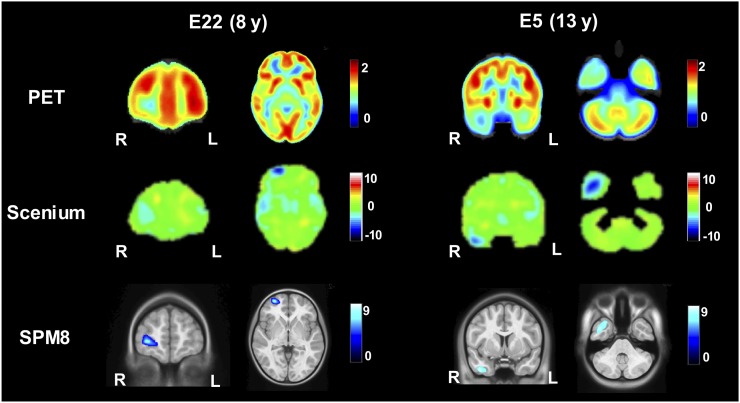
Comparison of voxelwise statistical maps of Scenium (*z* score) and SPM8 (*t* value). Two representative slices (from patients E22 and E5) approximately at same anatomic level were chosen, considering the different size of standard spaces within Scenium and SPM8.

Figure 3 compares the voxelwise statistical maps obtained for two representative patients. In both patients, SPM8 correctly detected the presumed epileptogenic areas located in the right frontal lobe and right temporal lobe, respectively, as reported by the MDT diagnosis. These regions were also highlighted in the *z* score maps from Scenium. However, despite being visible on the maps, these regions did not reach the significance threshold of less than −2 SDs in the region-of-interest–based statistical tables, considered for clinical reporting. Indeed, for patient E22, the most hypometabolic region was found in the left temporal lobe, with −3 SDs, whereas the right frontal lobe had only −0.3 SDs. In the case of patient E5, the most hypometabolic region, with −2.3 SDs, was placed in the left parietal region, whereas the right temporal lobe resulted in −1.0 SDs.

### SPM8 Pipeline: Validation with Postsurgical Seizure-Free Patients

**FIGURE 4.**
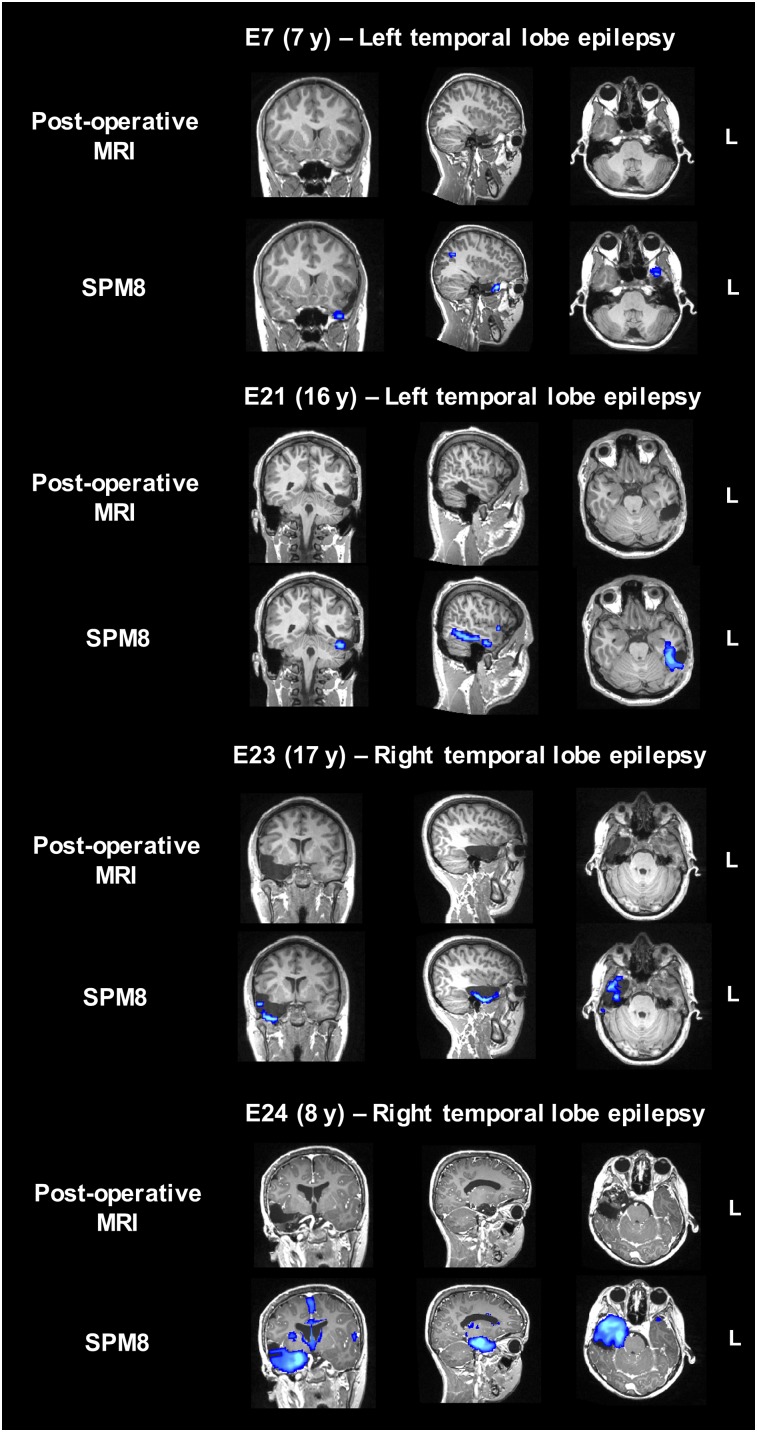
Presurgical SPM8-based statistical maps overlaid on coregistered postsurgical MRI scans for four representative seizure-free patients (patients E7, E21, E23, and E24).

To further validate the ad hoc pipeline based on SPM8, we selected a subgroup of patients who underwent surgical resection of the presumed focus with positive outcome (Engel class I, Fig. 1). Lateralization and localization of the most significant cluster to the resected area were assessed using postoperative MRI scans or surgery reports (when postsurgical scans were not available). For lateralization and localization, SPM8 showed a sensitivity of 67% and 67%, respectively, for the younger patient group (*n* = 3) and 80% and 70%, respectively, for the older patient group (*n* = 10). The SPM8-based statistical maps overlaid on the postoperative MRI scans with the resection cavity clearly visible are shown in Figure 4 for four representative patients. The area reported as most significant was correctly colocalized with the resected area in all patients. For patient E7, the small hypometabolic area at the edge of the temporal lobe was shifted to the border of the brain during the registration steps to bring the map back to native space.

To further quantify the degree of overlap, the percentage of voxels falling within the resection cavity was computed. This analysis was performed on only 5 of 10 patients with postsurgery MRI scans; one patient had thermal ablation and hence the resection area was not present, whereas in the other four seizure-free patients the statistical cluster did not overlap with the resected area. The percentage of voxels falling within the resection cavity was more than 50% in most patients (Table 2). For patient E7, the lower percentage was due to suboptimal registration, which caused the statistical map to shift to the edge of the brain (Fig. 4). Hence, fewer voxels fell within the resection cavity. For patient E21, the cluster containing the maximum as represented on the SPM8 map only partially covered the lesion (Fig. 4), leading to a lower overall percentage within the resected area (34%).

**TABLE 2:** Proportion of Voxels Falling Within Resection Cavity

Patient	Voxels falling within resection cavity
E7	5%
E16	68%
E21	34%
E23	71%
E24	53%

### SPM8 Pipeline: Non–Seizure-Free Patients

**FIGURE 5.**
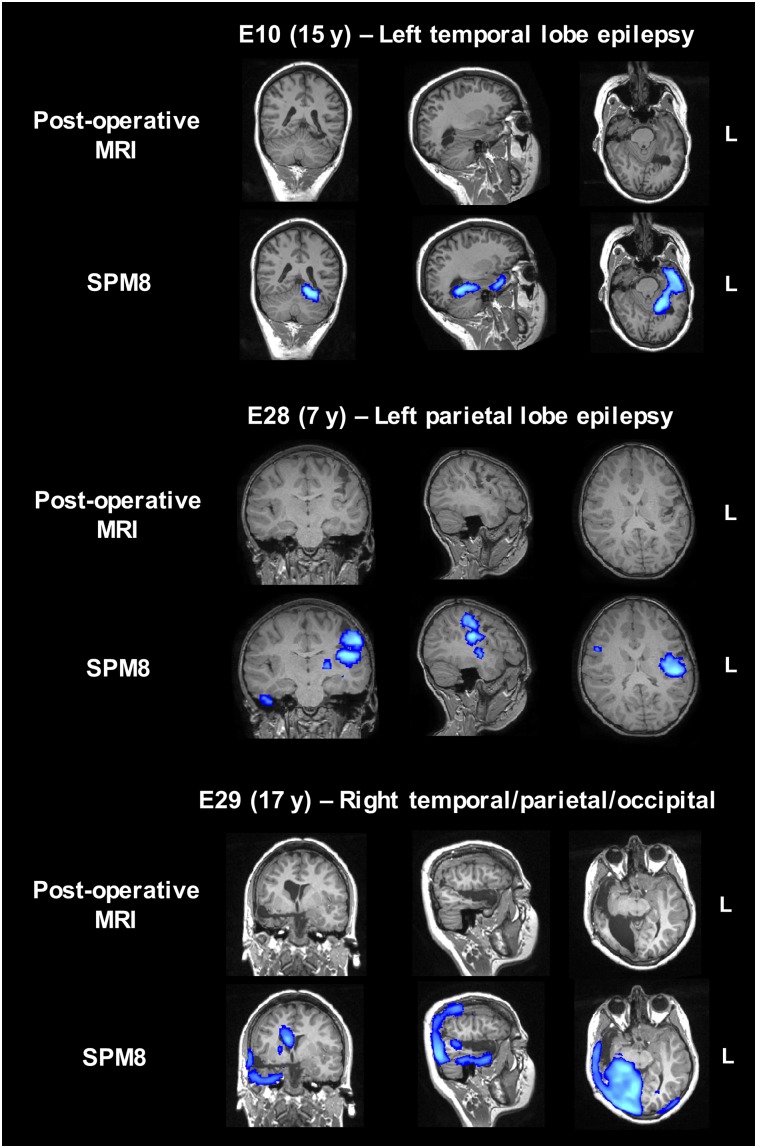
Presurgical SPM8-based statistical maps overlaid on coregistered postsurgical MRI scans for three representative non–seizure-free patients (patients E10, E28, and E29).

Five patients were not seizure-free after surgery (Fig. 1). In three of these patients, SPM8-based statistical maps reported a more diffuse hypometabolism that extended farther than the resected area. In the remaining two patients, the cluster containing the maximum colocalized with the area resected. Figure 5 shows the results of the SPM8 analysis for three non–seizure-free patients. For patients E10 and E29, SPM8 reported a more extensive hypometabolism than the area resected, which might relate to a non–seizure-free outcome. Patient E29 is of particular note, given the presence of right ventricle dilatation, highlighting a more profound pathology that is not likely to be resolved by the focus resection. Patient E28 represents a case in which the statistical map correctly colocalized to the resected area, but the patient was not seizure-free after surgery.

## DISCUSSION

The aim of this work was to provide a reliable observer-independent pipeline for the analysis of pediatric ^18^F-FDG PET scans, as part of presurgical planning in epilepsy. Two age-specific ^18^F-FDG PET templates in standard pediatric space (as opposed to adult MNI space) were created, representing children between 6–9 and 10–20 y old. Voxelwise statistics were performed using the commercial software Scenium (using an adult normative dataset in MNI space) and an SPM8 pipeline (using pediatric templates and datasets). Our results show improved agreement with the MDT diagnosis for both age ranges using the customized SPM8 pipeline, with a sensitivity of about 70% for the localization of hypometabolic areas on pediatric ^18^F-FDG PET scans. Overall, we showed that it is essential to use age-matched ^18^F-FDG PET templates and databases to achieve reliable and accurate results.

Computer-assisted reporting is on the rise in fields of medicine such as breast cancer detection (14), lung cancer screening (15), and coronary artery disease identification (16), in which the use of computer-assisted systems together with visual assessment has been shown to reduce diagnostic error and false-negative rates (15,16). The adoption of these computer systems has shown potential not only to shift from double- to single-image readings (14) but also for use in emergency room settings, where automatic systems can flag a possibly critical condition for immediate intervention (16). In the field of neurology, several commercial software applications have been made available by vendors to aid visual reporting of brain ^18^F-FDG PET scans as part of presurgical planning (12). Additionally, previous studies adopted the open-source software SPM for automatic voxelwise analysis of ^18^F-FDG PET data and for informing presurgical planning (2,17,18). In most of these analyses, adult standard space and non–age-matched control groups were used (12,17,18), mainly because of ethical constraints that prevent the acquisition of a normative pediatric PET dataset.

Only two studies reported the creation of an in-house pediatric ^18^F-FDG PET template obtained using a pseudocontrol group of epilepsy patients with normal findings on ^18^F-FDG PET scans (2,7). However, the procedure used to build these templates was not described well enough to allow the analyses to be reproduced. In this work, we outline a detailed two-step registration procedure to create two pediatric templates using a dataset of children without central nervous system diseases. Our results justified the need for a second registration step that was essential to improve the alignment of the control scans to the pediatric standard space, especially for the younger group. In this way, more scans could be included to derive the final template. Our pediatric normative dataset was significantly larger than those used in previous studies (112 subjects, as compared with 24 (2) and 21 (7)), allowing the creation of templates representing two age groups: 6–9 y and 10–20 y. This age stratification is more accurate than having a unique template representing a wider age range, as it is known that metabolism and brain size change considerably between 6 and 20 y of age (2). Ideally, groups having even smaller age ranges could be considered (and below 6 y), but such groups were not possible in our study because of the low number of younger controls, which were grouped to guarantee the creation of a reliable template and enough power for the subsequent voxelwise statistics. In the future, the reference database can be extended by including ^18^F-FDG PET images from different scanners to mitigate biases introduced by specific acquisition and reconstruction parameters and to make the database more widely applicable.

The agreement of the SPM8 pipeline results with the MDT diagnosis was compared with Scenium, a commercial software package currently used to aid ^18^F-FDG PET reporting in the clinic. Previous studies compared the voxelwise results with the visual assessment of ^18^F-FDG PET data (2,7,17). In this study, Scenium was used as a benchmark to assess the performance of Scenium to detect hypometabolic areas on ^18^F-FDG PET scans and to investigate whether the presented SPM8 pipeline and age-specific templates improve visual assessment of pediatric ^18^F-FDG PET data in the clinic. Increased agreement with the MDT diagnosis was found using SPM8 with pediatric templates, thereby supporting the potential of this pipeline as a tool for pediatric ^18^F-FDG PET reporting. Sensitivity values were in the range of those reported previously for seizure-free patients (2,17,18). For the three non–seizure-free patients, we reported a more diffuse hypometabolism than in the resected area. Hence, we speculate that a more extensive resection, if supported by other assessments and imaging modalities, might have provided a better outcome. With this work, we aim to outline a reliable and robust method that can be applied to a larger postsurgery patient group to compute metrics to investigate the usefulness of this technique (i.e., positive and negative predictive values) and to assess the difference in outcomes between resecting more than 50% and less than 50% of the significantly hypometabolic area (19,20).

There are limitations to using semiautomated reporting methods. Voxelwise analyses, despite avoiding obvious user dependencies, can introduce new biases due to differences between the cohort of interest and the reference database. This limitation can be mitigated by accurately assessing each registration step in the pipeline, but it cannot be completely avoided, as normalization to a common space is fundamental for subsequent statistical analysis. Another limitation of this study regards the use of SPM8, which is not Food and Drug Administration–approved, as opposed to Scenium. However, the latter, being commercial software, provides limited flexibility in both the analysis and the sharing of the database (a license is needed). This limitation hampered the complete matching of the steps between the two pipelines. The data were smoothed with a 6-mm and 12-mm kernel in SPM8 and Scenium, respectively. However, we could not modify the smoothing kernel inbuilt in Scenium, and we decided to use a different smoothing size in SPM8 to follow the kernel size recommended in the literature (e.g., 2 or 3 times the voxel size). Additionally, larger smoothing kernels, such as the fixed 12-mm kernel used in Scenium, increase spatial artifacts and can decrease sensitivity in detecting epileptogenic areas (17). The cerebellum gray matter uptake was used to perform intensity normalization in SPM8, because the glucose uptake of this region is usually minimally affected in epilepsy (3). Hence, its mean value was used for intraparticipant intensity normalization, rather than using the mean brain uptake (used by Scenium), as this can lead to biases in the detection of hypometabolic regions, especially if the patient has a global low baseline metabolism.

## CONCLUSION

We have shown that with readily available data and applications, it is possible to provide a more accurate user-independent semiquantitative method of assessing the ^18^F-FDG PET scans of patients under 18 y old by creating two age-specific templates and using appropriate pediatric control databases. This analysis can be useful to complement the current visual assessment of ^18^F-FDG PET images performed in the clinic.

## DISCLOSURE

This work is supported by the EPSRC-funded UCL Centre for Doctoral Training in Medical Imaging (EP/L016478/1), the Department of Health’s NIHR-funded Biomedical Research Centre at UCLH and GOSH, by the MRC, and by the ALSAC. No other potential conflict of interest relevant to this article was reported.
