# Supplementary material for: Age-Specific 18F-FDG Image Processing Pipelines and Analysis Are Essential for Individual Mapping of Seizure Foci in Pediatric Patients with Intractable Epilepsy
Source: J Nucl Med. 2018 Oct;59(10):1590–6. doi: 10.2967/jnumed.117.203950 (PMC6167536; doi:10.2967/jnumed.117.203950)
Supplement: Supplementary file 1 [file jnm203950SupplementalData.pdf]

Supplemental Table 1: Demographic and clinical information of the patient population analysed in this work. The histological information is reported only when available after surgical resection.

Subject	Sex	Age at PET (years)	Location Defined by Multidisciplinary Meeting	MRI lesion	Histology
E1	M	14	Right frontal (cingulate)	Subtle cortical dysplasia in right cingulate gyrus	Presumed dysplasia type 2. Small sample making diagnosis complex
E2	M	10	Reported as normal	Left hemisphere is smaller (particularly temporal lobe)	
E3	M	8	Right frontal	Venous abnormalities. No focal cortical dysplasia	
E4	M	9	Right frontal and left temporal	Negative	
E5	F	13	Right anterior temporal	Slightly reduced brain volume, negative otherwise	Reactive changes and evidence of acquired cortical and white matter damage. No evidence of an underlying lesion to explain epilepsy
E6	M	8	Right temporal	Negative	
E7	M	7	Left temporal	Left mesial temporal sclerosis	Hippocampal sclerosis, granular cellular dispersion
E8	F	15	Right anterior temporal	Negative	The tissue shows reactive changes many of which are related of the placement of intracranial electrodes. No diagnostic features.
E9	F	16	Left frontal	Negative	
E10	F	15	Left temporal	Lesion in left mesial temporal lobe (extend to left hippocampus and parahippocampal gyrus)	Parahippocampal gyrus: low grade glioneuronal tumour favouring a ganglioglioma (WHO grade I)
E11	F	17	Multifocal (bilateral temporal)	Some poverty of grey-white matter differentiation in the right temporal lobe (uncertain significance)	

E12	F	16	Right frontal	Blurring of the cortical and white matter junction involving the left occipital temporal gyrus and the inferior parietal lobule, for cortical dysplasia. No other abnormalities (2009 and negative on later scans)	Reactive changes and some acquired damage (invasive monitoring). No diagnostic evidence of a malformation or underlying pathology
E13	F	17	Left anterior temporal	Suspicious area of possible abnormal sulcation with prominent cortical vein in the left parietal lobe (possible dysplasia)	Non-specific features that are frequent in patients with seizures (white matter vascular changes and Chasiln's changes). No specific diagnostic features
E14	F	6	Multifocal	Loss of brain volume (pronounced in cerebellum) Negative	
E15	M	11	Reported as normal	Negative	
E16	F	11	Left anterior temporal	Prominence in the right choroid fissure, no specific features of mesial temporal sclerosis. No lateralising or localising features	Temporal lobectomy, hippocampal sclerosis (ILAE type 1) and granular cell dispersion
E17	F	9	Left frontal	Left cerebral volume loss. Left hemisphere posterior frontal and perisylvian volume loss (Rasmussen's encephalitis)	
E18	M	12	Left frontal	Focal cortical dysplasia (IIB) left middle frontal gyrus	Focal cortical dysplasia type 2b
E19	M	6	Right parietal	Left frontal lobe possible focal cortical lesion, small foci of deep white matter signal change in the peritrigonal regions (mature gliotic scars)	No characteristics of focal cortical dysplasia, small scar. Further examination requested
E20	F	9	Right parietal (patchy)	Asymmetry of grey matter differentiation and white matter signal, poorer in the right frontal lobe. No convincing evidence of focal cortical dysplasia	
E21	M	16	Left posterior temporal	Malformation of cortical development focal cortical dysplasia left temporal sulcus with features of focal cortical dysplasia 2b (signal abnormality extending to the ventricular margins)	Focal cortical dysplasia type 2b

E22	M	8	Right frontal	Right frontal lobe lesion (middle frontal gyrus). Abnormal signal within the cortex along the superior longitudinal fissure and widening of the sulcus	
E23	F	17	Right anterior temporal	Right mesial temporal sclerosis	Hippocampal sclerosis (ILAE type 1) with granule cell dispersion
E24	F	8	Right temporal	Mature brain injury to the adjacent right temporal lobe and frontal lobe	Cortical and white matter calcification, granule cell dispersion, Hippocampal sclerosis (ILAE type 2)
E25	M	17	Right temporal	Negative	
E26	F	17	Left temporal lobe	Left mesial parietal cortical dysplasia and diffuse left temporal lobe abnormalities (left mesial temporal lobe sclerosis)	Hippocampal sclerosis (ILAE type 1), granular cell dispersion and mossy fibre sprouting
E27	M	10	Left posterior insula and post-central)	Negative	Thermal coagulation
E28	F	7	Left parietal operculum and insula	Abnormal cortical folding in the left parietal operculum, plus blurring of grey-white matter junction which is consistent with cortical dysplasia. More extensive bilateral malformation	Focal cortical dysplasia type 2b
E29	F	16	Right temporal/parietal/occipital	Porencephalic dilatation of the right lateral ventricle with gliosis of the right occipital lobe. The right cerebral hemisphere is smaller than the left	Segmental neural loss and gliosis in the hippocampus
E30	M	17	Left anterior temporal	Left cerebral hemisphere is smaller than the right (particularly temporal lobe). Blurring of the cortical grey and subcortical white matter in the anterior temporal pole of the left with associated hippocampal sclerosis (might be focal cortical dysplasia)	Hippocampal sclerosis (ILAE type 1) with granule cell dispersion and mossy fibre sprouting
E31	F	12	Multifocal	Mild while matter bulk with mild prominence of the lateral ventricles and a thin corpus callosum, in keeping with developmental delays. Small nonspecific left thalamic scar	
E32	F	10	Left precuneus/parietal	Mature region of damage with cortical and white matter scarring in the left mesial parietal lobe which	

				is nonspecific but could be old infract. Few smaller foci of signal abnormality in the deep white matter of the right frontal lobe, these are not specific
E33	M	15	Left occipital	Area of blurring of the grey/white matter junction in the left mesio-occipital region (focal cortical dysplasia)
E34	M	6	Multifocal	Negative
E35	F	16	Multifocal	Small lesion in the medial aspect of the left thalamus, which is non-specific
E36	F	9	Right frontoparietal	Blurring of the grey-white matter interfaces involving the right frontal lobe and including the frontal opercular region, most marked in the superior frontal gyrus
E37	M	10	Left post central gyrus around motor cortex	Negative
E38	M	16	Right frontal	Negative
E39	F	17	Patchy right hemisphere	Negative
E40	M	6	Right temporal	Patchy, posterior, in temporal pole myelination not complete on both sides, more on the right. Abnormalities in white matter
E41	M	16	Multifocal (bilateral hypometabolism)	Signal in the right temporal white matter and right hippocampus are slightly brighter than the left side, but this can be seizure related. No cortical malformation, no changes in hippocampi, no abnormal diffusion in brain parenchyma
E42	M	14	Multifocal (bilateral hypometabolism)	Right hemisphere is slightly smaller than the left. Immaturity in myelin maturation in the right hemisphere, with poor grey-white matter differentiation (anterior right temporal lobe and anterior parts of the perisylvian cortex)
E43	F	9	Multifocal (left lateralisation)	Negative

E44	F	9	Left temporal	Negative
E45	F	16	Multifocal (bilateral temporal)	Motor cortices a bit abnormal, subtle right motor cortex strip
E46	F	16	Right hemisphere	Enlarged ventricles and swollen right hemisphere. Non-progressive atrophy of the right frontal lobe and the right operculum

Table 2: Clinical diagnosis of each patient and corresponding area highlighted by Scenium and SPM8 pipelines. The cases of disagreement are reported in red.

Subj.	Location Defined by Multidisciplinary Meeting	Results Scenium	Results SPM8
**E1**	Right frontal (cingulate)	Right parietal	Posterior right frontal
**E2**	Reported as normal	Multifocal	Right frontal
**E3**	Right frontal	Right frontal	Right frontal
**E4**	Right frontal and left temporal	Bilateral frontal	Right frontal and left temporal
**E5**	Right anterior temporal	Left central/parietal	Right anterior temporal
**E6**	Right temporal	Right temporal	Left temporal
**E7**	Left temporal	Left frontal	Left temporal
**E8**	Right anterior temporal	Right temporal	Right temporal
**E9**	Left frontal	Left frontal	Left frontal + others Parietal
**E10**	Left temporal	Left temporal	Left temporal
**E11**	Multifocal (bilateral temporal)	Multifocal	Multifocal
**E12**	Right frontal	Left temporal	Left temporal
**E13**	Left anterior temporal	Left temporal	Left temporal
**E14**	Multifocal	Multifocal	Multifocal
**E15**	Reported as normal	Left temporal	Left temporal posterior
**E16**	Left anterior temporal	Left temporal	Left temporal
**E17**	Left frontal	Left frontal	Left frontal
**E18**	Left frontal	Left frontal	Normal
**E19**	Right parietal	Left frontal	Left operculum/temporal
**E20**	Right parietal (patchy bilateral)	Patchy bilateral	Patchy bilateral
**E21**	Left posterior temporal	Left temporal	Left temporal
**E22**	Right frontal	Left temporal	Right frontal
**E23**	Right anterior temporal	Right temporal	Right temporal
**E24**	Right temporal	Right temporal	Right temporal

**E25**	Right temporal	Right temporal	Right temporal
**E26**	Left temporal	Left temporal	Left temporal
**E27**	Left posterior insula and post-central	Left central/	Left posterior insula and post-central
**E28**	Left parietal operculum and insula	Left frontal	Left parietal operculum and insula
**E29**	Right temporal/parietal/occipital	Right occipital parietal	Right occipital temporal (wider)
**E30**	Left anterior temporal	Left parietal	Left temporal (wider)
**E31**	Multifocal	Multifocal	Multifocal
**E32**	Left precuneus/parietal	Left parietal	Left precuneus/parietal
**E33**	Left occipital	Normal	Left occipital
**E34**	Multifocal (right hemisphere)	Multifocal	Multifocal (right hemisphere)
**E35**	Multifocal	Multifocal	Multifocal
**E36**	Right frontoparietal	Right frontoparietal	Right frontoparietal
**E37**	Left post central gyrus (motor)	Left frontal	Left post central gyrus
**E38**	Right frontal	Normal	Left frontal
**E39**	Patchy right hemisphere	Patchy right	Patchy right hemisphere
**E40**	Right temporal	Right temporal	Right temporal
**E41**	Bilateral hypometabolism	Bilateral hypometabolism	Bilateral hypometabolism
**E42**	Bilateral hypometabolism	Bilateral hypometabolism	Bilateral hypometabolism
**E43**	Left hemisphere (not clear)	Left hemisphere	Left hemisphere
**E44**	Left temporal	Normal	Left posterior/parietal
**E45**	Bilateral temporal	Bilateral temporal	Bilateral temporal
**E46**	Right hemisphere	Right hemisphere	Right hemisphere
